# Waning of vaccine-induced measles antibodies and population immunity gaps in the post-elimination era: a multicenter seroepidemiological study in China

**DOI:** 10.3389/fimmu.2026.1919103

**Published:** 2026-08-18

**Authors:** Wen Wang, Zhisheng Chen, Hanwen Zhang, Jinting Xie, Yuanyuan Zhu, Guzel Nurpulat, Xiang Sun

**Affiliations:** 1 Department of Rheumatology and Immunology, Jiangsu Province (Suqian) Hospital, Suqian, Jiangsu, China; 2 Ili Prefectural Center for Disease Control and Prevention, Xinjiang, China; 3 Department of Microbiology and Immunology, School of Biomedical Sciences, McGill University, Montreal, QC, Canada; 4 Xuzhou Center for Disease Control and Prevention, Xuzhou, Jiangsu, China; 5 Department of Expanded Program on Immunization, Jiangsu Provincial Center for Disease Control and Prevention, Nanjing, Jiangsu, China

**Keywords:** antibody waning, internal migration, measles elimination, propensity score matching, vaccine-induced immunity

## Abstract

**Background:**

Despite sustained high measles-containing vaccine (MCV) coverage, maintaining population immunity after measles elimination remains challenging. This study aimed to characterize measles antibody persistence, identify immunity gaps, and evaluate factors associated with seronegativity in China.

**Methods:**

A multicenter cross-sectional serological survey was conducted among 2,083 participants from Jiangsu Province and Xinjiang Uygur Autonomous Region during 2024–2025. Measles-specific IgG antibodies were measured using ELISA, with seropositivity defined as IgG ≥200 mIU/mL. Restricted cubic spline models were used to assess antibody kinetics after excluding individuals without documented vaccination, those sampled within one month after vaccination, and potential primary vaccine failures. Multivariable logistic regression and propensity score matching were performed to evaluate factors associated with seronegativity.

**Results:**

Overall measles IgG seropositivity was 84.59% (95% CI: 82.98–86.08%), with a GMT of 482.48 mIU/mL. Seropositivity varied significantly by region, ethnicity, age, and vaccination history. Antibody levels showed a non-linear decline with increasing time since vaccination, and each additional year since the last vaccination increased seronegativity risk (AOR = 1.11, 95% CI: 1.07–1.15). Children aged 8–17 months had the lowest seropositivity (28.13%), indicating a potential susceptibility window. Although migrants had higher seropositivity than permanent residents (93.04% vs. 83.90%), migration status was not independently associated with seronegativity after adjustment.

**Conclusions:**

High MCV coverage does not necessarily ensure sustained population immunity in the post-elimination era. Waning humoral immunity, delayed vaccination, and heterogeneous vaccination histories contribute to residual susceptibility, highlighting the need for immunity-based surveillance and targeted interventions.

## 1 Background

Measles remains one of the most contagious human viral pathogens globally (1). Despite decades of widespread deployment of measles-containing vaccine (MCV) driving dramatic reductions in global measles-related mortality, progress toward the World Health Organization’s (WHO) Immunization Agenda 2030 (IA2030) goal of global measles elimination has stalled markedly over the past decade (2). The theoretical herd immunity threshold for measles is estimated at 92%–95%, corresponding to an intrinsic basic reproduction number (R_0_) of 12–18; yet recurrent large outbreaks in many high-vaccine-coverage nations demonstrate sustained population susceptibility due to two dominant drivers: primary vaccination failure and progressive waning of vaccine-derived humoral immunity with time post-vaccination (3, 4). Natural boosting from wild measles circulation, once a critical supplementary source of population-level immunity, has been largely eliminated in most middle- and high-income settings, further accelerating population-level antibody decay and raising outbreak vulnerability worldwide (5).

China achieved indigenous wild-type measles elimination in 2012 via sustained routine childhood immunization and targeted supplementary immunization activities (SIAs) (6), resulting in historically low annual measles notifications. Nevertheless, the country’s measles epidemiological profile has shifted toward a classic bimodal case distribution, with disease concentrated in unimmunized infants aged <8 months and children experiencing delayed dose-2 administration (8–17 months), as well as older adolescents/adults with waned vaccine immunity. This underscores the critical need to dissect childhood immunity gaps using granular age stratifications aligned with vaccine administration milestones (7, 8). In this post-elimination epidemiological setting, the absence of endemic wild-virus exposure removes natural immune reinforcement, meaning population measles IgG levels follow a continuous declining trajectory as time elapses after MCV inoculation, gradually expanding the pool of susceptible individuals and posing persistent regional outbreak risks (9).

Regional and ethnic disparities alongside population mobility constitute key unresolved challenges to national immunity evaluation in China. Existing domestic population serosurveys are mostly restricted to single-city or provincial sampling, failing to capture cross-regional and multi-ethnic immunity heterogeneity across economically divergent regions. Eastern developed Jiangsu Province and multi-ethnic western Xinjiang Uygur Autonomous Region represent two highly contrasting demographic, geographic, and socio-economic archetypes in China, serving as an ideal dichotomous model to unpack such disparities. Jiangsu, an eastern coastal economic powerhouse, represents a densely populated, ethnically homogeneous area (exclusively Han Chinese in our cohort) where the primary public health challenge involves managing rapid urbanization and massive inflows of domestic migrants. Conversely, Xinjiang, a vast northwestern border region, is characterized by its sparse population and profound ethnic diversity (including Uyghur, Kazakh, and other minorities), which presents distinct challenges related to logistics over vast geographical distances, cultural/language barriers in healthcare delivery, and mobile pastoralist tracking. Of particular research controversy is the so-called migrant paradox: unadjusted crude analyses commonly report higher measles seroprevalence among internal migrants than local permanent residents, which is frequently attributed to the healthy migrant effect (10). However, such uncorrected comparisons are confounded by uneven age composition, divergent immunization access and socioeconomic gaps, leading to biased immunity estimates and hampering evidence-based immunization policy design (11). To date, few domestic serological studies have applied rigorous causal inference approaches to eliminate baseline imbalance between migrant and resident cohorts, nor have they quantitatively modelled the non-linear trajectory of long-term vaccine antibody waning across distinct populations.

Against this research gap, we conducted a cross-sectional seroepidemiological survey enrolling 2083 participants from Jiangsu and Xinjiang. We adopted restricted cubic spline (RCS) regression to quantify the non-linear decay kinetics of measles IgG antibodies over years post-vaccination, and implemented propensity score matching (PSM)-based causal analysis to balance confounding covariates and resolve the migrant immunity paradox. This study systematically evaluates the independent effects of geographic, ethnic, immunization and migration factors on population measles serostatus. Findings provide empirical evidence for targeted SIAs optimization in China and deliver a reference epidemiological model for middle-income countries experiencing rapid demographic transition and domestic population migration under global measles elimination efforts.

## 2 Methods

### 2.1 Study design and population

This cross-sectional multicenter seroepidemiological survey was conducted from January 2024 to December 2025 across two representative provincial-level regions of China: Jiangsu Province (economically developed eastern coastal region dominated by Han ethnicity) and Xinjiang Uygur Autonomous Region (ethnically diverse western border region with multi-ethnic settlements). These two regions were selected to establish a robust dichotomous model representing contrasting demographic, socio-economic, and healthcare delivery challenges in China. Jiangsu, a densely populated and ethnically homogeneous coastal province, faces public health challenges centered on rapid urbanization and managing massive influxes of inbound migrants. Conversely, Xinjiang is a vast, sparsely populated landlocked border region characterized by profound ethnic diversity (including Uyghur, Kazakh, and other minorities) and unique geographical barriers, making vaccine logistics and cultural communication key priorities. Stratified multi-stage cluster random sampling was strictly implemented following the national routine immunization sampling specification issued by China CDC.

Eligible permanent residents and cross-county internal migrants were randomly enrolled at the individual level from routine immunization registry rosters. Migration status was not used as a predefined sampling stratum or quota during recruitment; instead, it was determined according to the National Health Commission definition after enrollment. The final cohort consisted of 2083 participants, including 1925 permanent residents and 158 internal migrants. Internal migrants were defined according to the National Health Commission of China criteria as individuals residing outside their registered household county for ≥30 days without local household registration. No upper limit was applied for the duration of residence; individuals who had lived outside their registered household county for extended periods but retained their original household registration were classified as internal migrants.

#### 2.1.1 Sample size calculation

Based on previously published national measles seroprevalence data (expected positive rate p=85.0%), α=0.05 (Z1−α/2 = 1.96)), absolute permissible sampling error δ=2.0%, the baseline minimal sample size was calculated as (N=Z^2^×p×(1−p)/δ^2^). Design effect (DEFF = 1.5) was adopted referring to domestic multi-region immunization serosurvey literatures; an additional 10% sample reserve was added to compensate for blood sample failure and participant non-response, resulting in target enrollment 2042 participants. Final enrolled valid cohort contained 2083 participants, matching pre-calculated sample requirement.

#### 2.1.2 Inclusion criteria

(1) Complete documented MCV immunization records (injection dates and total doses retrievable from provincial immunization information management system, which were verified against the official national and regional MCV schedules outlined in **Supplementary Materials** (**Supplementary Table 1**); (2) Integral demographic information including age, gender, ethnic category and migration status; (3) Available peripheral venous blood specimens for quantitative IgG detection.

#### 2.1.3 Exclusion criteria

Participants with congenital/acquired severe immunodeficiency, blood transfusion or intravenous blood product exposure within 3 months before sampling, acute febrile infectious diseases at blood collection day were excluded to avoid interference on endogenous antibody level.

### 2.2 Laboratory assays

Peripheral venous blood 2 mL was collected from each enrolled participant without anticoagulant; serum was separated within 4 h post blood collection, aliquoted and preserved at -20°C for centralized batch detection. Quantitative measles-specific IgG antibody concentration was measured using a commercially standardized indirect ELISA kit (SERION ELISA classic, Institut Virion/Serion GmbH, Würzburg, Germany, Batch No. EP0180, EP0262 and EP0202), all experimental manipulations strictly followed manufacturer’s operating manual. Test results were uniformly expressed in milli-international units per milliliter (mIU/mL). In line with the consensus guidelines and standard clinical seroprotection criteria issued by the WHO, an antibody titer > 200 mIU/mL was defined as seropositive.

### 2.3 Variable definitions and core derived indicators

Two analytical endpoints were set for subsequent statistics: continuous outcome (log_10_-transformed measles IgG geometric titer) and binary outcome (seropositive vs seronegative for logistic regression). In accordance with WHO standard criteria and kit specifications, measles IgG antibody concentration ≥200 mIU/mL was defined as seropositive, while concentration <200 mIU/mL was defined as seronegative, which served as the primary binary endpoint for multivariable logistic regression modeling.

Core independent exposure variable: migration status (internal migrant/permanent resident). Confounding covariates were predefined based on clinical immunology and measles epidemiological evidence: age (categorized into six granular groups to align with vaccination milestones: < 8 months, 8–17 months, 18 months–3 years, 4–6 years, 7–14 years, and ≥15 years), gender, geographic region (Jiangsu/Xinjiang), ethnicity (Han, Uyghur, Kazakh, other ethnic minorities), cumulative documented MCV vaccination doses (0/1/2/≥3 doses).

Key derived variable: Interval since last valid MCV vaccination, calculated as continuous months between the administration date of final documented MCV dose and specimen collection date; calendar month difference was corrected by Gregorian calendar to eliminate uneven days per-month bias. All variable coding rules were pre-specified in study statistical analysis plan before database lock.

### 2.4 Statistical analysis

All statistical computational frameworks were executed using R software (Version 4.2.1), with the formal threshold for two-tailed statistical significance established at P<0.05. To accommodate the severely skewed, non-normal distribution of antibody titers, raw metrics were log10-transformed to calculate Geometric Mean Titers (GMT) with 95% confidence intervals (CIs). To compare differences in GMT and seropositivity rates across demographic and vaccination subgroups, independent-sample t-tests (or one-way ANOVA for multi-group comparisons) and Pearson’s chi-square tests (*χ*^2^) were performed, respectively, while overall and subgroup seropositivity rates were estimated alongside 95% CIs utilizing the Wilson score interval method to ensure statistical rigor. To map the continuous trajectory of antibody waning without making arbitrary linear assumptions, RCS with four knots positioned at the 5th, 35th, 65th, and 95th percentiles were fitted within an ordinary least squares framework. To model the pure physiological trajectory of vaccine-induced antibody decay, individuals with zero documented MCV doses, individuals within the immediate 1-month post-vaccination window, and individuals exhibiting primary vaccine failure (seronegative within 3 months post-vaccination) were strictly excluded from the restricted cubic spline analysis. Then, followed by a multivariable logistic regression model incorporating prespecified epidemiologically relevant variables and potential confounders, including vaccination history, time since vaccination, demographic characteristics, geographic region, ethnicity, and migration status. to derive adjusted odds ratios (AOR) for predictors of seronegativity. To rigorously evaluate the causal relationship between migration status and measles seropositivity, a Directed Acyclic Graph (DAG) was constructed to delineate confounding versus mediating pathways (**Supplementary Figure 1**). Age, sex, region, and ethnicity were identified as baseline confounders. MCV doses and time since last vaccination were recognized as potential mediators on the causal pathway from migration status to seropositivity. To estimate both the total causal effect and the controlled direct effect of migration status, two complementary PSM specifications were executed: A Total Effect Model matching strictly on pre-exposure baseline confounders (age, sex, region, ethnicity); and A Controlled Direct Effect Model additionally matching on MCV doses to assess whether serological decay differs between migrants and permanent residents who received identical vaccine regimens. A 1:1 nearest-neighbor matching algorithm without replacement (caliper = 0.02) was implemented for both specifications.

## 3 Results

### 3.1 Baseline demographic profiles and spatial-ethnic heterogeneity in seroprotection

A total of 2,083 participants were included in the analysis, with a median age of 6.20 years (IQR: 3.33,12.30). Among them, 1,170 (56.17%) participants were from Xinjiang and 913 (43.83%) from Jiangsu. Males accounted for 53.38% (1,112/2,083) of participants. The majority were Han Chinese (57.90%), followed by Kazakh (18.15%), Uyghur (15.17%), and other ethnic groups (8.79%). Most participants were permanent residents (92.41%), while 7.59% were migrants. For measles-containing vaccine (MCV) doses, 74.41% received 2 doses, 9.99% 0 doses, 2.02% 1 dose, and 13.59% ≥3 doses (**Table 1**).

**Table 1:** Demographic characteristics of participants.

Variables	Categories	n (%)/(IQR)
Total Participants		2083 (100.00%)
Age (years)		6.20 (3.33, 12.30)
Region	Xinjiang	1170 (56.17%)
	Jiangsu	913 (43.83%)
Sex	Male	1112 (53.38%)
	Female	971 (46.62%)
Ethnic group	Han	1206 (57.90%)
	Kazakh	378 (18.15%)
	Uyghur	316 (15.17%)
	Others	183 (8.79%)
Migration status	Permanent residents	1925 (92.41%)
	Migrants	158 (7.59%)
(MCV doses	0 dose	208 (9.99%)
	1 dose	42 (2.02%)
	2 doses	1550 (74.41%)
	≥3 doses	283 (13.59%)

**Figure 1:**
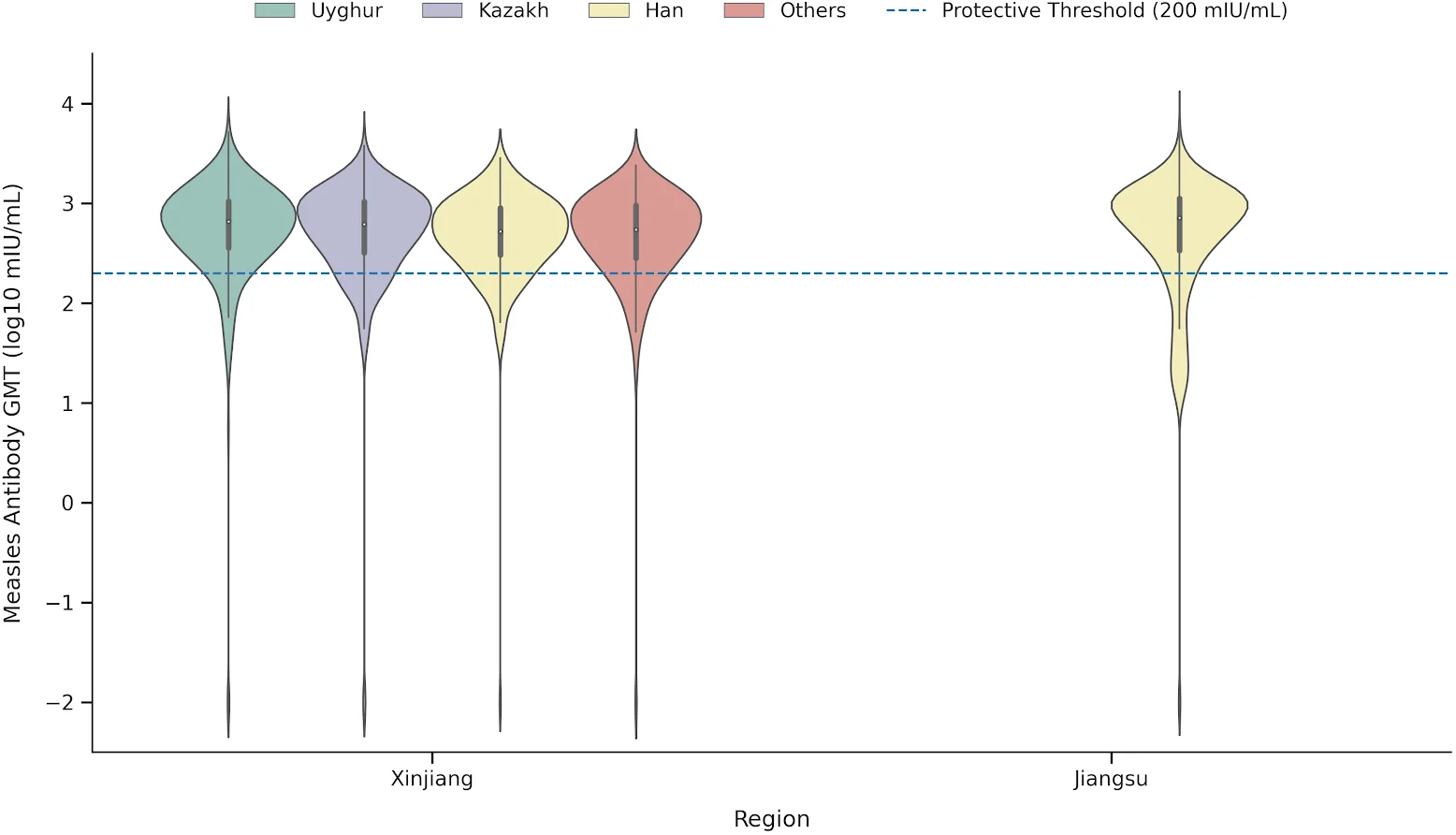
Stratified distribution of measles IgG titers across regions and ethnicities. Grouped boxplots demonstrating the interaction of geography and ethnicity on antibody levels. The horizontal blue dashed line marks the clinical protective threshold (200 mIU/mL, log10 = 2.30).

Overall, measles IgG seropositivity was 84.59% (95% CI: 82.98%–86.08%), with a GMT of 482.48 mIU/mL (95% CI: 455.63–510.90). Seropositivity differed significantly by region, ethnicity, and migration status. Participants from Xinjiang showed higher seropositivity than those from Jiangsu (86.41% vs. 82.26%, P = 0.011). Uyghur participants had the highest seropositivity (90.19%), while Han participants had relatively lower seropositivity (82.75%) (*P* = 0.012). Migrants showed higher seropositivity and GMT levels compared with permanent residents (93.04% vs. 83.90%, *P* = 0.003) (**Figure 1**).

Given that children younger than 3 years include different immunological stages according to the national MCV schedule, this group was further stratified. Infants younger than 8 months had a seropositivity of 47.62% (GMT = 130.32mIU/mL), reflecting waning maternal antibodies before vaccination. Children aged 8–17 months showed the lowest seropositivity (28.13%, GMT = 60.29 mIU/mL), representing the interval before completion of the vaccination schedule. In contrast, children aged 18–36 months showed high seropositivity (96.35%, GMT = 909.46mIU/mL) after routine vaccination. Vaccination history was strongly associated with measles immunity. Participants with two or more MCV doses had substantially higher seropositivity and GMT levels than those without vaccination (*P* < 0.001) (**Table 2**).

**Table 2:** Comparison of measles antibody levels by characteristics.

Variable	N	Seropositivity rate n (%) (95% CI)	*χ2*/*P*	GMT (mIU/mL) (95% CI)	*F*/*P*
Region					
Xinjiang	1170	1011 (86.41%, 84.33%–88.26%)	6.47/0.011	495.95 (462.76–531.53)	1.14/0.285
Jiangsu	913	751 (82.26%, 79.64%–84.60%)		465.74 (423.25–512.50)	
Age group					
<8 months	84	40 (47.62%, 36.60–58.82)	325.08/<0.001	130.32 (96.15–176.59)	113.46/<0.001
8–17 months	96	27 (28.13%, 19.84–38.25)		60.29 (39.82–91.30)	
18 months-3 years	219	211 (96.35%, 92.96–98.34)		909.46 (814.73–1015.20)	
4–6 years	602	582 (96.68%, 94.92–97.84)		769.03 (722.84–818.17)	
7–14 years	689	582 (84.47%, 81.58–86.98)		439.03 (401.46–480.12)	
≥15 years	387	320 (82.69%, 78.60–86.13)		446.94 (396.47–503.84)	
Sex					
Male	1,112	934 (83.99%, 81.72%–86.03%)	0.56/0.455	480.36 (446.43–516.86)	0.03/0.872
Female	971	828 (85.27%, 82.90%–87.36%)		484.91 (443.34–530.38)	
Ethnic group					
Han	1206	998 (82.75%, 80.52%–84.78%)	11.00/0.012	467.72 (432.64–505.64)	1.10/ 0.348
Uyghur	316	285 (90.19%, 86.41%–93.00%)		536.96 (466.69–617.80)	
Kazakh	378	323 (85.45%, 81.54%–88.65%)		500.47 (439.50–569.90)	
Others	183	156 (85.25%, 79.38%–89.66%)		456.38 (384.52–541.67)	
Migration status					
Permanent residents	1925	1615 (83.90%, 82.19%–85.47%)	8.67/0.003	470.73 (443.38–499.78)	8.71/0.003
Migrants	158	147 (93.04%, 87.96%–96.07%)		651.40 (540.74–784.70)	
MCV doses					
0 dose	208	87 (41.83%, 35.33%–48.62%)	325.08/<0.001	108.91 (82.73–143.37)	113.46/<0.001
1 dose	42	36 (85.71%, 72.16%–93.28%)		370.91 (195.97–702.03)	
2 doses	1550	1382 (89.16%, 87.52%–90.61%)		575.37 (544.03–608.52)	
≥3 doses	283	257 (90.81%, 86.88%–93.65%)		571.09 (519.72–627.53)	
Total	2083	1762(84.59%, 82.98–86.08)		482.48 (455.63–510.90)	

### 3.2 Non-linear kinetics of antibody decay and multivariable risk factors for seronegativity

**Figure 2:**
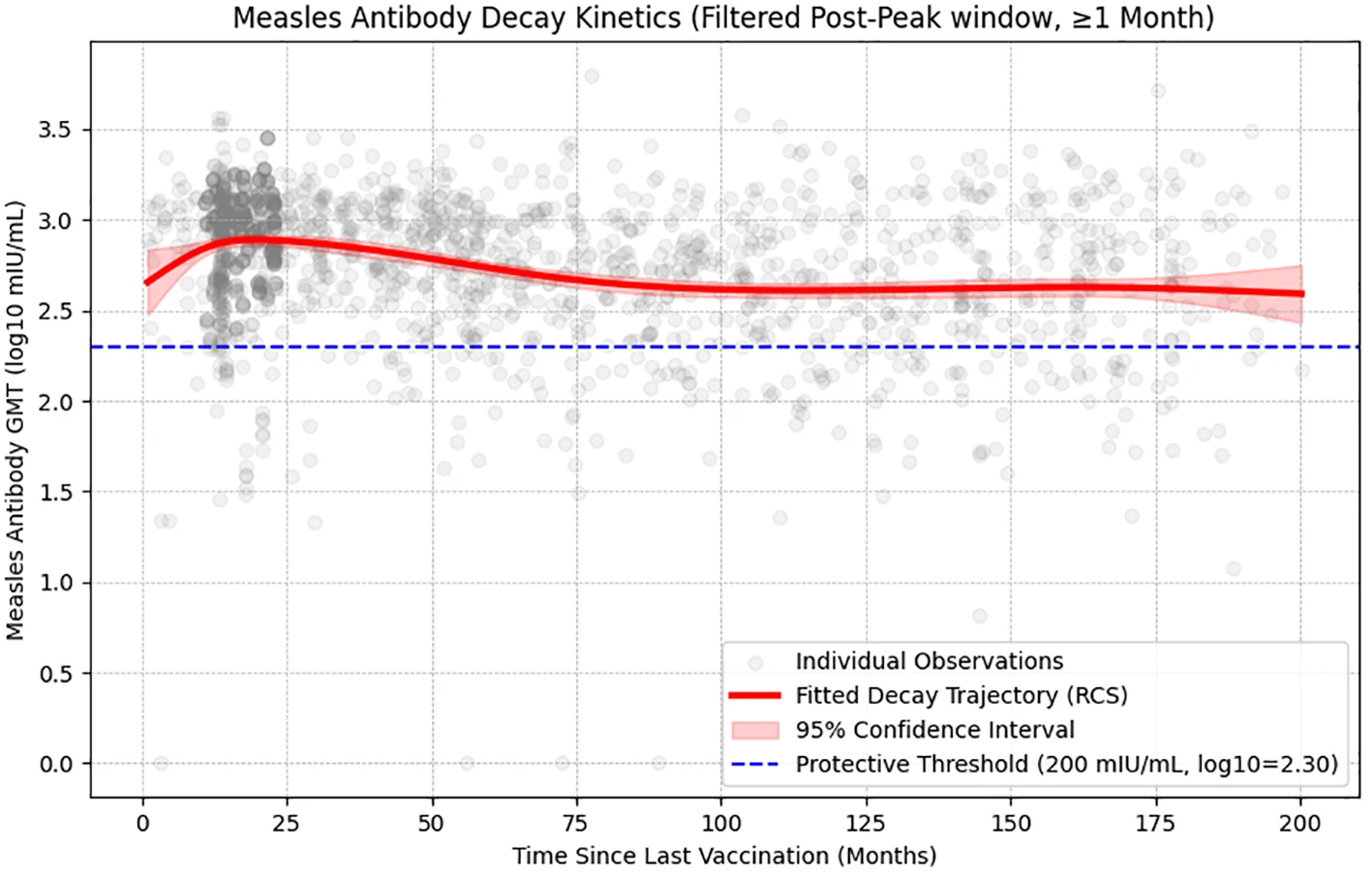
Non-linear kinetic trajectory of vaccine-induced antibody decay via restricted cubic splines (RCS). The solid red line models the smooth continuous kinetics of log-transformed measles GMT over the time interval since the last vaccination, accompanied by its 95% shaded confidence bands (knots enforced at the 5th, 35th, 65th, and 95th percentiles).

To model the pure physiological waning of vaccine-induced immunity, a restricted cubic spline (RCS) model was fitted to 1815 individuals, excluding those with zero MCV doses or within the immediate post-vaccination window (<1 month) (**Figure 2**). Controlling for these early immune confounding factors eliminated the artifactual initial rise. The revised curve demonstrated a clear biphasic decay trajectory: antibody levels peaked and plateaued within the first 24 months, underwent a steady decline between 24 and 75 months, and finally decelerated into a prolonged, stable plateau up to 200 months post-vaccination. Although the population-level fitted trajectory remained above the clinical protective threshold (200mIU/mL) throughout the 16-year continuous registry, the lower bound of the 95% confidence interval approached the threshold at the tail end (>175 months), indicating potential vulnerabilities to long-term waning in a post-elimination setting.

**Figure 3:**
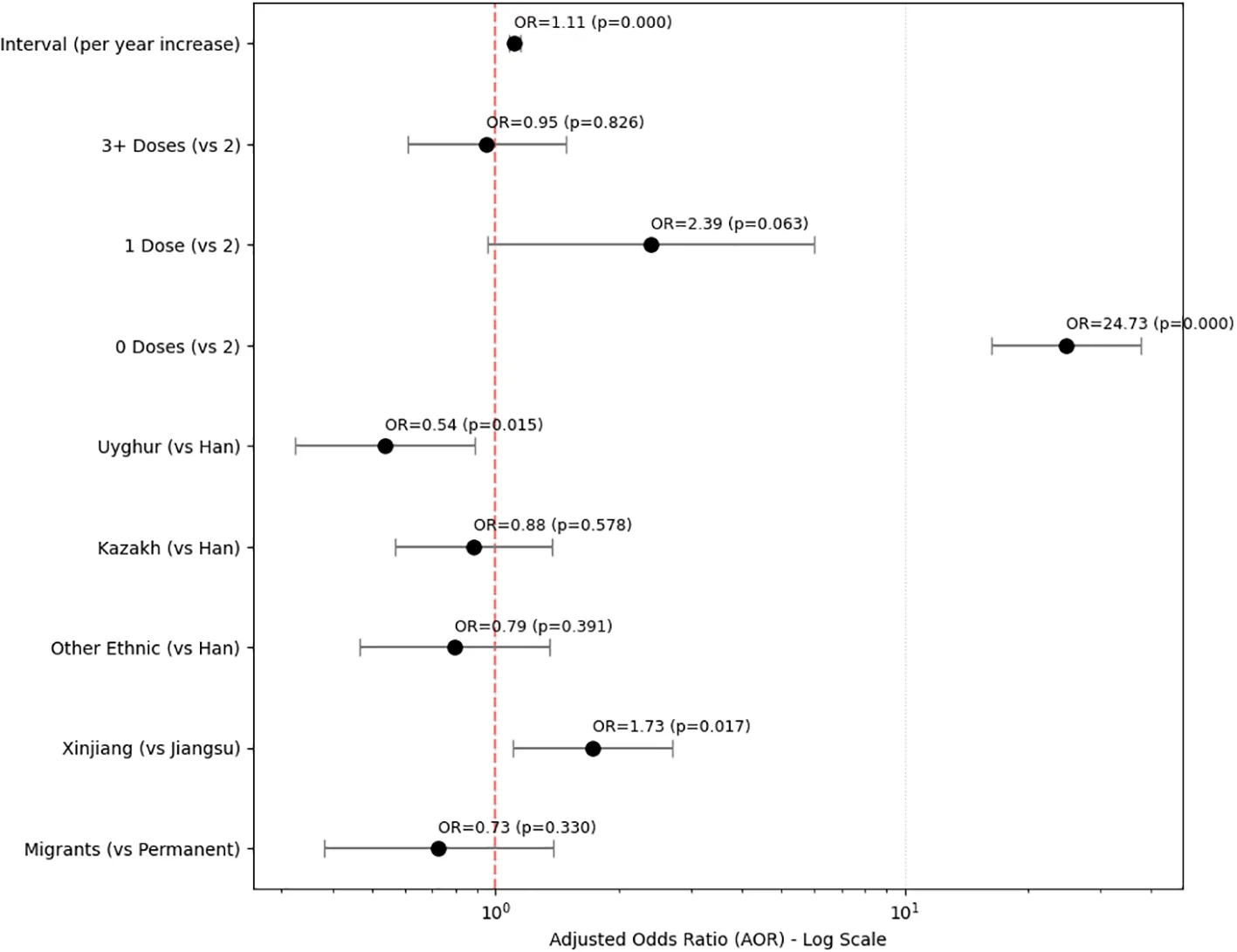
Multivariable forest plot of parametric predictors driving measles seronegativity. Derived from fully adjusted multivariable logistic regression modeling; independent markers are plotted on a logarithmic scale with their corresponding 95% confidence intervals.

Multivariable logistic regression (**Figure 3**; **Table 3**) identified time since vaccination as a key continuous risk factor: each 1-year increase elevated seronegativity risk by 11.08% (AOR = 1.11, 95%CI:1.07-1.15, *P* < 0.001). 0 dose of MCV conferred drastically higher risk (AOR = 24.73, 95%CI:16.26-37.61, *P* < 0.001) versus 2 doses. Even after adjusting for these structural histories and temporal waning, region and ethnicity remained independent biological drivers; residents in Xinjiang experienced 1.73 times the odds of falling into seronegativity relative to Jiangsu (AOR = 1.73, 95% CI:1.10-2.71, *P* = 0.017), whereas the Uyghur ethnicity was confirmed as a profound independent protective factor, incurring only 53.58% of the seronegativity risk faced by the Han population (AOR = 0.54, 95% CI:0.32-0.89, *P* = 0.015). Crucially, in this fully adjusted regression model, the crude advantage of migration status evaporated entirely (*P* = 0.33), demonstrating that migration per se does not govern antibody maintenance.

**Table 3:** Multivariable logistic regression of risk factors for measles seronegativity.

Variables (seronegativity <200 mIU/mL)	Reference	AOR (95% CI)	P-value
Interval since vaccination (per year increase)	Continuous	1.11 (1.07-1.15)	< 0.001
MCV Doses	2 doses	–	–
0 dose		24.73 (16.26-37.61)	< 0.001
1 dose		2.39 (0.95-6.00)	0.063
≥3 doses		0.95 (0.61-1.48)	0.826
Region	Jiangsu	1.73 (1.10-2.71)	0.017
Ethnic Group	Han	–	–
Uyghur		0.54 (0.32-0.89)	0.015
Kazakh		0.88 (0.57-1.37)	0.578
Migration Status	Permanent	0.73 (0.38-1.38)	0.330

Model adjusted for geographic region (Jiangsu vs. Xinjiang), ethnicity (Han, Uygur, Kazakh, Others), number of MCV doses (0/1/2/), time elapsed since last vaccination (years), age (years), and sex. Dependent variable: Measles seronegativity (IgG <200 mIU/mL).

Subgroup analysis revealed critical immunity gaps (**Table 4**). In Xinjiang, migrant Han individuals had low GMT (347.82 mIU/mL, seropositivity: 81.82%) versus minority migrants (GMT:704.25 mIU/mL, seropositivity: 95.00%). In Jiangsu, migrant children aged 7–14years exhibited a low seropositivity of 0.00% (GMT: 76.51 mIU/mL, N = 2), though this cell is limited by a small sample size, significantly lower than local permanent peers (76.27%, GMT: 392.34 mIU/mL, *P* < 0.05).To align with the NIP milestones, we further granularized the young pediatric cohort in Jiangsu (**Table 2**): infants aged <8 months exhibited profound vulnerability with a seropositivity of 47.62%due to maternal antibody decay; toddlers aged 8–17 months showed a moderate seropositivity of 28.13% driven by immunization delays.

**Table 4:** Serological metrics within granular risk subgroups.

Region	Subgroup characteristics	Sample (n)	Seropositivity (%)	GMT (mIU/mL)
Xinjiang	Migrants - Han	11	81.82%	347.82
	Migrants - Minorities	100	95.00%	704.25
Jiangsu	Migrants - (7-14 years)	2	0.00%	76.51
	Permanent - (7-14 years)	118	76.27%	392.34
	Permanent - (0-3 years)	64	67.19%	277.95

### 3.3 Propensity score matching and sensitivity analysis

To eliminate baseline demographic imbalances and evaluate potential selection bias between permanent residents (n=1925) and internal migrants (n=158), a 1:1 nearest-neighbor PSM algorithm without replacement was performed using a tight caliper of 0.02. Guided by our causal framework (**Supplementary Figure 1**), two complementary PSM specifications were executed to evaluate both the Total Causal Effect and the Controlled Direct Effect of migration status on measles seronegativity (**Table 5**).

**Table 5:** Baseline characteristics and measles seronegativity before and after propensity score matching (PSM).

Variable	Unmatched cohort	Model 1: total effect model	Model 2: controlled direct effect model
Demographic balance			
Age (years, Mean ± SD)	9.82 ± 6.14 vs. 8.45 ± 5.92(SMD=0.228)	8.45 ± 5.92 vs. 8.51 ± 5.88(SMD=0.010)	8.45 ± 5.92 vs. 8.48 ± 5.90(SMD=0.005)
Sex (Male, %)	52.41% vs. 54.43% (SMD=0.041)	54.43% vs. 53.80% (SMD=0.013)	54.43% vs. 54.43% (SMD=0.000)
Region (Xinjiang, %)	55.17% vs. 68.35% (SMD=0.273)	68.35% vs. 68.35% (SMD=0.000)	68.35% vs. 68.35% (SMD=0.000)
Ethnicity (Han, %)	58.18% vs. 51.90% (SMD=0.126)	51.90% vs. 52.53% (SMD=0.013)	51.90% vs. 51.90% (SMD=0.000)
MCV Doses (2 Doses, %)	74.49% vs. 73.42% (SMD=0.024)	73.42% vs. 75.32% (SMD=0.043)	73.42% vs. 73.42% (SMD=0.000)
Primary outcome			
Seronegativity Rate (%)	15.74% (303/1925) vs. 11.39% (18/158)	15.82% (25/158) vs. 11.39% (18/158)	15.19% (24/158) vs. 11.39% (18/158)
Effect estimates			
Odds Ratio (OR/AOR)	Crude OR=0.69 (95% CI:0.41–1.14)	Total OR=0.68 (95% CI:0.36–1.31)	Direct AOR=0.72 (95% CI:0.37–1.38)
P-value	P=0.147	P=0.252	P=0.316

SMD < 0.10 indicates adequate balance between matched cohorts.

Model 1 assesses the Total Causal Effect by matching strictly on baseline confounders (age, sex, region, ethnicity) without adjusting for downstream vaccination history.

Model 2 assesses the Controlled Direct Effect by matching on baseline confounders plus MCV dose history (0/1/2/≥3 doses) to evaluate whether antibody levels differ given identical vaccination regimens.

#### 3.3.1 Model 1

Total Causal Effect Model (Primary Pre-exposure Matching) In Model 1, propensity scores were estimated by matching strictly on baseline pre-exposure confounders (age, sex, region, and ethnicity), leaving potential downstream mediators (MCV doses and time since last vaccination) unadjusted. Matching yielded 158 balanced pairs (N = 316). Standardized mean differences (SMDs) for all matched baseline covariates fell below 0.10, indicating excellent covariate balance between cohorts. In this total effect analysis, measles seronegativity rates were 11.39% among internal migrants and 15.82% among permanent residents. Migration status exerted no statistically significant total effect on measles seronegativity risk (OR = 068, 95% CI:0.36-1.31, *P* = 0.252).

#### 3.3.2 Model 2

Controlled Direct Effect Model (Sensitivity Analysis) In Model 2, propensity scores were estimated by matching on baseline confounders plus MCV dose history (0/1/2/≥3 doses) to isolate the direct effect of migration status under equalized vaccination exposure. All 158 internal migrants were successfully matched 1:1 with permanent residents (N = 316). Following matching, seronegativity rates remained equivalent between migrants (11.39%, 18/158) and permanent residents (15.19%, 24/158). Holding vaccination coverage constant, internal migration status demonstrated no direct association with measles seronegativity (AOR = 0.72, 95% CI:0.37-1.38, *P* = 0.316).

## 4 Discussion

This multicenter cross-sectional serological study provides evidence regarding measles population immunity and antibody persistence in two distinct epidemiological settings in China. Despite sustained reported coverage of MCV1 and MCV2 above 95% in Jiangsu and Xinjiang, the overall measles IgG seropositivity in our study population was 84.59%, below the estimated 92–95% immunity level required to interrupt transmission (2, 12). These findings indicate that high administrative vaccination coverage does not necessarily reflect uniformly maintained population immunity (13–15), and that susceptible groups may persist even in highly vaccinated populations (6, 7).

Following the interruption of endemic measles transmission, opportunities for natural boosting have substantially decreased. Therefore, population immunity increasingly depends on the persistence of vaccine-induced protection (16). In our study, measles IgG concentrations declined progressively with increasing time since vaccination, demonstrating measurable waning of humoral immunity in the post-elimination era (9). In the restricted analysis excluding individuals without documented vaccination, samples collected shortly after vaccination, and potential primary vaccine failures, antibody levels showed a non-linear decline pattern characterized by a rapid early decrease followed by a slower attenuation phase, consistent with previous reports of vaccine-induced antibody persistence (17, 18). Importantly, declining antibody concentrations should be interpreted as waning measurable humoral immunity rather than definitive secondary vaccine failure. Although antibody levels are an important marker of protection, measles vaccine-induced immunity also involves immunological memory, including memory B-cell and T-cell responses (19). Therefore, individuals with antibody levels below the conventional serological threshold may still retain some degree of immune protection. Future studies integrating cellular immunity assessments and breakthrough infection surveillance are needed to clarify the clinical relevance of long-term antibody decline. Nevertheless, in the absence of natural boosting, continuous monitoring of population immunity and maintaining high two-dose vaccination coverage remain essential for sustaining measles elimination.

Beyond overall antibody dynamics, our findings revealed substantial heterogeneity in measles immunity across geographic and demographic groups. Jiangsu, despite its higher socioeconomic development and healthcare accessibility, showed lower seropositivity and greater variability in antibody levels than Xinjiang. This difference suggests that population immunity is influenced not only by vaccination coverage but also by historical exposure patterns, demographic characteristics, and regional immunization practices. The higher seropositivity observed among Uyghur participants should also be interpreted cautiously, as it may reflect differences in vaccination history, historical exposure, or regional immunization implementation rather than ethnicity itself. Age-specific analyses further identified potential periods of increased susceptibility. The lower seropositivity among infants younger than 8 months likely reflects the expected decline of maternally derived antibodies before routine vaccination (20). More importantly, the reduced seropositivity among children aged 8–17 months suggests a potential susceptibility window between loss of maternal antibodies and establishment of vaccine-induced immunity. This finding highlights the importance of timely MCV1 administration and continuous evaluation of vaccination strategies to minimize immunity gaps during early childhood.

Migration status also requires careful interpretation in the context of measles immunity. Unlike the general “healthy migrant effect,” measles serological status is primarily determined by vaccination access, vaccine uptake, and previous exposure history. Migrants may have lower immunity because of incomplete vaccination opportunities or healthcare barriers, but they may also show higher seropositivity due to greater historical exposure, different vaccination practices in their regions of origin, or vaccination before or after migration (21). In our study, migrants initially showed higher seropositivity than permanent residents; however, migration status was not independently associated with measles seronegativity after adjustment for demographic characteristics and vaccination-related factors (10). These findings suggest that migration itself is unlikely to determine measles immunity, whereas differences in vaccination and exposure histories are more important contributors.

This study has several limitations. First, the cross-sectional design prevented direct evaluation of individual antibody trajectories, and longitudinal studies are needed to validate antibody decay patterns. Second, ELISA IgG measurements represent a surrogate marker of humoral immunity and do not fully capture neutralizing antibodies or cellular immune responses (22, 23). Third, detailed migration histories and geographic origins were unavailable, limiting evaluation of the influence of origin-specific vaccination and exposure patterns. Finally, some subgroup analyses involved limited sample sizes and should therefore be interpreted cautiously.

In conclusion, high reported MCV coverage does not necessarily guarantee optimal population-level measles immunity. Residual susceptibility appears to result from the combined effects of waning measurable humoral immunity, delayed vaccination, and heterogeneous vaccination histories. Migration status itself does not appear to determine antibody persistence; rather, observed differences among migrant populations are likely explained by differences in vaccination and exposure patterns. These findings support a shift toward immunity-based strategies integrating routine immunization, serological surveillance, and targeted interventions to maintain measles elimination.

## Data Availability

The raw data supporting the conclusions of this article will be made available by the authors, without undue reservation.
