# Supplementary material for: Waning of vaccine-induced measles antibodies and population immunity gaps in the post-elimination era: a multicenter seroepidemiological study in China
Source: Front Immunol. 2026 Aug 18;17:1919103. doi: 10.3389/fimmu.2026.1919103 (PMC13529708; doi:10.3389/fimmu.2026.1919103)
Supplement: Supplementary file 1 [file Supplementaryfile1.docx]

**Figure S1. Directed Acyclic Graph (DAG) illustrating causal pathways between migration status and measles seropositivity.** **Unadjusted backdoor paths are blocked by matching on baseline confounders (age, sex, region, ethnicity). Matching on MCV doses isolates the controlled direct effect by holding vaccine dose exposure constant across matched pairs.**

Table S1. Official Measles-Containing Vaccine (MCV) Immunization Schedules in China

| Category | Vaccine Type | Target Age | Target Disease Coverage | Funding |
| --- | --- | --- | --- | --- |
| EPI (National Free Program) | MMR (Measles-Mumps-Rubella) | 1st Dose: 8 months  2nd Dose: 18 months | Measles, Mumps, Rubella | Fully funded by the government (Free) |
| Historical EPI (Pre-June 2020) | MR (Measles-Rubella)MMR (Measles-Mumps-Rubella) | 1st Dose: 8 months (MR)  2nd Dose: 18–24 months (MMR) | Measles, Rubella (D1)Measles, Mumps, Rubella (D2) | Fully funded by the government (Free) |
| Non-EPI (Self-pay Options) | Imported MMR (e.g., Priorix) | Same milestones or used for private catch-up / travel | Measles, Mumps, Rubella | Out-of-pocket payment by guardians |
